# Pulling cylindrical particles using a soft-nonparaxial tractor beam

**DOI:** 10.1038/s41598-017-00735-2

**Published:** 2017-04-05

**Authors:** Andrey Novitsky, Weiqiang Ding, Maoyan Wang, Dongliang Gao, Andrei V. Lavrinenko, Cheng-Wei Qiu

**Affiliations:** 1 DTU Fotonik, Technical University of Denmark, Kongens Lyngby, DK-2800 Denmark; 2 Department of Theoretical Physics and Astrophysics, Belarusian Stateparticle University, Minsk, 220030 Belarus; 3 Physics Department, Harbin Institute of Technology, Harbin, 150001 China; 4 Department of Electrical and Computer Engineering, National University of Singapore, Singapore, 117576 Singapore; 5 College of Physics, Optoelectronics and Energy, Soochow University, Suzhou, 215006 China

## Abstract

In order to pull objects towards the light source a single tractor beam inevitably needs to be strongly nonparaxial. This stringent requirement makes such a tractor beam somewhat hypothetical. Here we reveal that the cylindrical shape of dielectric particles can effectively mitigate the nonparaxiality requirements, reducing the incidence angle of the partial plane waves of the light beam down to 45° and even to 30° for respectively dipole and dipole-quadrupole objects. The optical pulling force attributed to the interaction of magnetic dipole and magnetic quadrupole moments of dielectric cylinders occurs due to the TE rather than TM polarization. Therefore, the polarization state of the incident beam can be utilized as an external control for switching between the pushing and pulling forces. The results have application values towards optical micromanipulation, transportation and sorting of targeted particles.

## Introduction

Tractor beams have recently drawn great attention of researchers owing to intriguing and sometimes counterintuitive physics behind them^1–11^. Electromagnetic^1–4^ and acoustic^5, 6^ wave tractor beams can possess continuous^1–3^ and discrete propagation invariance^4, 12, 13^, being able to pull an object over a long distance. Backward or pulling forces imposed by a continuous propagation-invariant tractor beam (single tractor beam, e.g., Bessel beam) are nonconservative forces. They emerge as a result of interaction of particle’s multipole moments, the most contribution being usually introduced by the dipole moments. The far-field indicator of pulling forces is large forward scattering, that is, the enhanced forward field momentum^8, 9^. Recent prediction of the negative torque^10^ and experiments on tractor beams^11^ stimulate further investigations of physics of tractor beams and their applications in optical manipulation^14–25^.

In order to produce a tractor beam exerting an optical pulling force, there are generally three different options. The first one is to employ a special kind of an incident light wave, such as a nonparaxial Bessel beam^1, 2, 7^, interfering multiple paraxial Bessel beams^4^, or properly designed superposition of multiple plane waves^3^. The second option is to impose restrictions on the background medium. Recently, it has been reported that complex structured dielectric^9, 26^ and metallic^27^ backgrounds are beneficial for the optical pulling force due to the forward light momentum amplification. The third approach is to apply restrictions on the object. When a light beam is absorbed by a lossy object, both the energy and momentum are transferred to the object, exerting naturally the pushing force due to the momentum conservation^28^. In this connection, when loss is ‘reversed’, an object with optical gain may generate an optical pulling force^29^, since it can greatly amplify the number of forward-scatterred photons.

**Figure 1:**
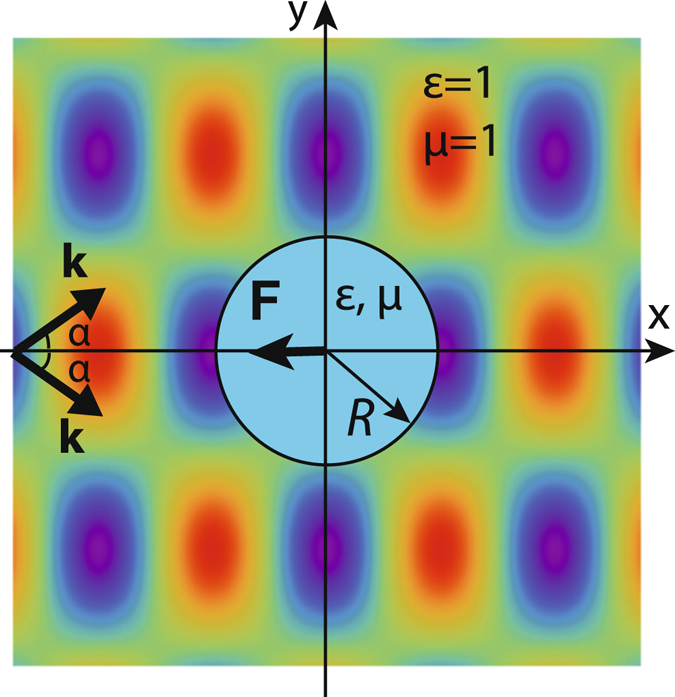
Continuous propagation-invariant light beam exerts pulling force *F* < 0 on a cylindrical object. The interference electric field pattern from a couple of TE-polarized plane waves (see equation (1)) is shown as the background.

In this article we deal with the continuous propagation-invariant tractor beams (nonparaxial superpositions of plane waves). Nonmagnetic objects in the Rayleigh approximation are always pushed by the continuous propagation-invariant light^7, 30^. Bigger spherical beads in the dipolar approximation have both electric and magnetic dipole moments and require *α* ≥ 60°, where *α* is the angle between the optical axis *x* and wavevectors **k** of the partial plane waves of the light beam (see Fig. 1). Angle *α* can be further reduced for bigger particles due to the interaction of the dipole and quadrupole moments, but still it is beyond the experimental possibilities of conventional optics in the case of Bessel tractor beams. Here we show that angle *α* can be decreased by shaping the particle in a specific way. Optical pulling forces exerting on cylindrical particles are first revealed by numerical modeling and then justified by closed-form calculations. Curiously, the discrete propagation-invariant beams have no limitations on angle *α* ^13^. They can be employed in the Rayleigh approximation for attracting even tiny particles.

## Results

Let us consider an infinitely long dielectric cylinder of radius *R* and permittivity $\varepsilon =\varepsilon ^{\prime} +i\varepsilon ^{\prime\prime}$ in free space. It is illuminated by an incident monochromatic wave with the field strengths ${\bf{E}}(x,y)\,\exp (-{\rm{i}}\omega t)$ and ${\bf{H}}(x,y)\,\exp (-{\rm{i}}\omega t)$, where *ω* is the circular frequency. For the sake of simplicity we exploit a model of such a light beam as a couple of plane waves with the same longitudinal wavenumbers *k*_0_ *β* = *k*_0_ cos *α* (see Fig. 1):

1 $$\begin{array}{rcl}{\bf{E}}(x,y) & = & {{\rm{e}}}^{{\rm{i}}\beta {k}_{0}x}[-{\rm{i}}qB\,\sin (q{k}_{0}y){{\bf{e}}}_{x}+\beta B\,\cos (q{k}_{0}y){{\bf{e}}}_{y}+A\,\cos (q{k}_{0}y){{\bf{e}}}_{z}],\\ {\bf{H}}(x,y) & = & {{\rm{e}}}^{{\rm{i}}\beta {k}_{0}x}[{\rm{i}}qA\,\sin (q{k}_{0}y){{\bf{e}}}_{x}-\beta A\,\cos (q{k}_{0}y){{\bf{e}}}_{y}+B\,\cos (q{k}_{0}y){{\bf{e}}}_{z}],\end{array}$$

where *k*_0_ = *ω*/*c* is the vacuum wavenumber, *c* is the speed of light in free space, and $q={k}_{y}/{k}_{0}=\sqrt{1-{\beta }^{2}}=\,\sin \,\alpha$ is the normalized transverse wavenumber. Complex amplitudes *A* and *B* relate to TE (*E*_*x*_ = 0 for *B* = 0) and TM (*H*_*x*_ = 0 for *A* = 0) electromagnetic beams, respectively.

In closed-form calculations we deal with particle’s parameters that allow only the excitation of electric **p** and magnetic **m** dipole and electric ${\hat{q}}_{e}$ and magnetic ${\hat{q}}_{m}$ quadrupole moments in the dielectric cylinder. Their values per unit cylinder’s length in terms of the scattering Mie coefficients *a*_*m*_ and *b*_*m*_ read (see Methods)

2 $$\begin{array}{rcl}{\bf{p}} & = & {\hat{\alpha }}_{e}{\bf{E}},\\ {\bf{m}} & = & {\hat{\alpha }}_{m}{\bf{H}},\\ {\hat{q}}_{e} & = & \frac{\cos \,\mathrm{(2}\alpha )}{\cos \,\alpha }{q}_{xy}{E}_{y}({{\bf{e}}}_{x}\otimes {{\bf{e}}}_{y}+{{\bf{e}}}_{y}\otimes {{\bf{e}}}_{x}),\\ {\hat{q}}_{m} & = & \frac{\cos \,\mathrm{(2}\alpha )}{\cos \,\alpha }{m}_{xy}{H}_{y}({{\bf{e}}}_{x}\otimes {{\bf{e}}}_{y}+{{\bf{e}}}_{y}\otimes {{\bf{e}}}_{x}),\end{array}$$

where the quantity ${({\bf{a}}\otimes {\bf{b}})}_{ij}={a}_{i}{b}_{j}$ (*i* and *j* run from 1 to 3) stands for a dyad (external or tensorial product of two vectors),

3 $${\hat{\alpha }}_{e}={\alpha }_{e}^{\perp }(1-{{\bf{e}}}_{z}\otimes {{\bf{e}}}_{z})+{\alpha }_{e}^{\parallel }{{\bf{e}}}_{z}\otimes {{\bf{e}}}_{z},\,{\hat{\alpha }}_{m}={\alpha }_{m}^{\perp }(1-{{\bf{e}}}_{z}\otimes {{\bf{e}}}_{z})+{\alpha }_{m}^{\parallel }{{\bf{e}}}_{z}\otimes {{\bf{e}}}_{z}$$

and

4 $$\begin{array}{rcl}{\alpha }_{e}^{\perp } & = & \frac{2i{a}_{1}}{\pi {k}_{0}^{2}},\\ {\alpha }_{e}^{\parallel } & = & \frac{i{b}_{0}}{\pi {k}_{0}^{2}},\\ {\alpha }_{m}^{\perp } & = & \frac{2i{b}_{1}}{\pi {k}_{0}^{2}},\\ {\alpha }_{m}^{\parallel } & = & \frac{i{a}_{0}}{\pi {k}_{0}^{2}},\\ {q}_{xy} & = & -\frac{4{a}_{2}}{\pi {k}_{0}^{3}},\\ {m}_{xy} & = & -\frac{4{b}_{2}}{\pi {k}_{0}^{3}}.\end{array}$$

Mie coefficients *a*_*m*_ = *a*_−*m*_ and *b*_*m*_ = *b*_−*m*_ describing the scattered field from respectively TM- and TE-polarized plane waves^31^ equal

5 $$\begin{array}{rcl}{a}_{m} & = & \frac{\sqrt{\frac{\varepsilon }{\mu }}{J}_{m}^{^{\prime} }(\xi ){J}_{m}(n\xi )-{J}_{m}(\xi ){J}_{m}^{^{\prime} }(n\xi )}{\sqrt{\frac{\varepsilon }{\mu }}{H}_{m}^{\mathrm{(1)}^{\prime} }(\xi ){J}_{m}(n\xi )-{H}_{m}^{\mathrm{(1)}}(\xi ){J}_{m}^{^{\prime} }(n\xi )},\\ {b}_{m} & = & \frac{\sqrt{\frac{\varepsilon }{\mu }}{J}_{m}(\xi ){J}_{m}^{^{\prime} }(n\xi )-{J}_{m}^{^{\prime} }(\xi ){J}_{m}(n\xi )}{\sqrt{\frac{\varepsilon }{\mu }}{H}_{m}^{\mathrm{(1)}}(\xi ){J}_{m}^{^{\prime} }(n\xi )-{H}_{m}^{\mathrm{(1)}^{\prime} }(\xi ){J}_{m}(n\xi )},\end{array}$$

where *ξ* = *k*_0_ *R* is the dimensionless size parameter of the cylinder of radius *R*, *μ* is the magnetic permeability (in our case *μ* = 1), $n=\sqrt{\varepsilon \mu }$ is the refractive index, *J*_*m*_ (${H}_{m}^{(1)}$) and ${J}_{m}^{^{\prime} }$ (${H}_{m}^{\mathrm{(1)}^{\prime} }$) are the *m*-order Bessel functions (Hankel functions) of the first kind and their derivatives.

First we carry out numerical calculation of the time-averaged optical forces using the integration of Maxwell’s stress tensor over the cylinder interface with outer normal vector **n** as ${\bf{F}}=\int \hat{T}{\bf{n}}ds=\int {(\hat{T})}_{ij}{n}_{j}ds$ (summation over repeated index *j* = 1, 2, 3 is assumed). Time-averaged stress tensor $\hat{T}=(c/8\pi ){\rm{Re}}[{\bf{E}}\otimes {{\bf{E}}}^{\ast }+{\bf{H}}\otimes {{\bf{H}}}^{\ast }-({|{\bf{E}}|}^{2}+{|{\bf{H}}|}^{2})/2]$ depends on total electric **E** and magnetic **H** fields defined as the sum of the incident and scattered fields. We denote the force per unit cylinder’s length as **f**. The force is pulling, if its direction is opposite to the direction of propagation of the incident beam (see Fig. 1), that is, if *f*_*x*_ < 0.

**Figure 2:**
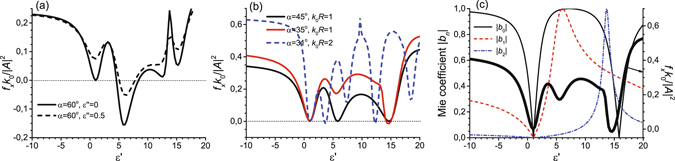
Optical force per unit cylinder length ${f}_{x}{k}_{0}/|{A}^{2}|$ vs. dielectric permittivity *ε*′ for (**a**) lossless and lossy cylindrical particles (*k*_0_ *R* = 1) and (**b**) different angles *α* of the oblique plane-wave incidence (*ε*″ = 0). (**c**) Absolute values of the Mie coefficients *b*_*n*_ responsible for the appearance of induced electric dipole (*b*_0_), magnetic dipole (*b*_1_) and magnetic quadrupole (*b*_2_) moments (the force shown on the right-hand axis corresponds to parameters *α* = 35°, *k*_0_ *R* = 1). Incident beam is TE-polarized (*B* = 0). The cylinder is nonmagnetic (*μ* = 1) and lossless.

We numerically study the optical forces exerted on dielectric cylinders illuminated by the incident TE-polarized electromagnetic beam (1). There are four parameters in such a system: dielectric permittivity *ε*′, loss parameter *ε*″, cylinder radius *R* and angle *α* (see Fig. 1). When angle *α* is sufficiently great (and the beam is strongly nonparaxial), the pulling force is mainly caused by the interaction of the electric |**p**| ~ *b*_0_ and magnetic |**m**| ~ *b*_1_ dipole moments (see the solid curve in Fig. 2(a)). Representing ${\alpha }_{e}^{\parallel }=|{\alpha }_{e}^{\parallel }|\,\exp (i{\varphi }_{0})$ and ${\alpha }_{m}^{\perp }=|{\alpha }_{m}^{\perp }|\,\exp (i{\varphi }_{1})$, the interaction term (recoil force) is proportional to $-{\rm{Re}}({\alpha }_{e}^{\parallel }{\alpha }_{m}^{\perp \ast })\sim -\cos ({\varphi }_{0}-{\varphi }_{1})$. The recoil force is negative and, therefore, may result in pulling, if $\cos ({\varphi }_{0}-{\varphi }_{1}) > 0$ or the dipoles are in phase. When the dipoles are in antiphase, the recoil force facilitates pushing. A nonzero loss coefficient *ε*″ increases the imaginary parts of polarizabilities. Subsequently, the conditions for getting the negative force are worsened (dashed curve in Fig. 2(a)).

When incident angle *α* diminishes to 45°, the pulling force owing to the interaction of dipoles cannot be realized (see Fig. 2(b)). Nevertheless, greater dielectric permittivities *ε* induce the non-vanishing magnetic quadrupole moment ${\hat{q}}_{m}$. And angle *α* suitable for pulling decreases down to 30° (see Fig. 2(b)). To check whether the magnetic quadrupole moment is responsible for the pulling effect, we plot the absolute values of the Mie coefficients |*b*_*n*_|, Fig. 2(c). As we mentioned above, the first two Mie coefficients are proportional to the dipole moments. According to equation (4) the magnetic quadrupole moment is proportional to the Mie coefficient *b*_2_. Interaction of multipole resonances, the first of which (magnetic dipole) is wide and the second one (magnetic quadrupole) is narrow, results in the Fano-like response similar to that of plasmonic particles^32, 33^. Thus, the negative force near *ε* = 14.5 is caused by interaction of the magnetic dipole and quadrupole moments.

**Figure 3:**
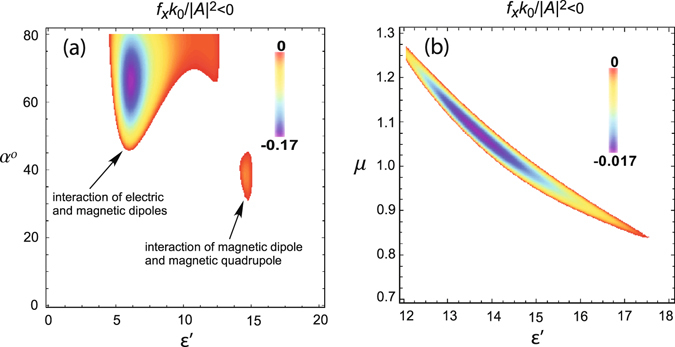
Dependence of the optical pulling force ${f}_{x}{k}_{0}/|{A}^{2}| < 0$ on (**a**) incident angle *α* and dielectric permittivity *ε*′ (*μ* = 1) and (**b**) magnetic permeability *μ* and permittivity *ε*′ (*α* = 35°). Incident wave is TE-polarized (*B* = 0). Cylinder parameters are *k*_0_ *R* = 1 and *ε*″ = 0.

The aforementioned decrease of incident angle *α* is demonstrated in a more vivid manner in Fig. 3(a). The dipole-dipole interaction is bottom limited by *α* = 45°. The region of the pulling force caused by the dipole-quadrupole interaction spreads from 30° to 45° and corresponds to the position of the magnetic quadrupole resonance. The dashed curve in Fig. 2(b) demonstrates the optical pulling force at the two positions of quadrupole resonances. The higher-order multipoles are excited as well, but they do not contribute into the pulling effect in this case.

In principle, higher-order multipoles could also produce a pulling force corresponding to *α* < 30°. However, the higher the order of a multipole, the narrower the resonance. This may manifest the problem with exploiting them, because the parameters should be selected with the high accuracy. Moreover, even a small loss can degrade the narrow resonance and, therefore, forbid the pulling force. So, it is impossible to loosely decrease *α* to any small value.

One can expect that magnetic properties of the cylinder facilitate stronger interaction between magnetic dipole and quadrupole, but it is not the case. The magnetic permeability shifts the position of the resonance, but does not extend much the range of the pulling force as it is shown in Fig. 3(b). It should be noted that the negative force exists both for paramagnetic and diamagnetic materials, and the force reaches the minimum value for paramagnetic substances. When the cylinder radius is enlarged, the number of resonances in the considered region of permittivities *ε*′ increases.

**Figure 4:**
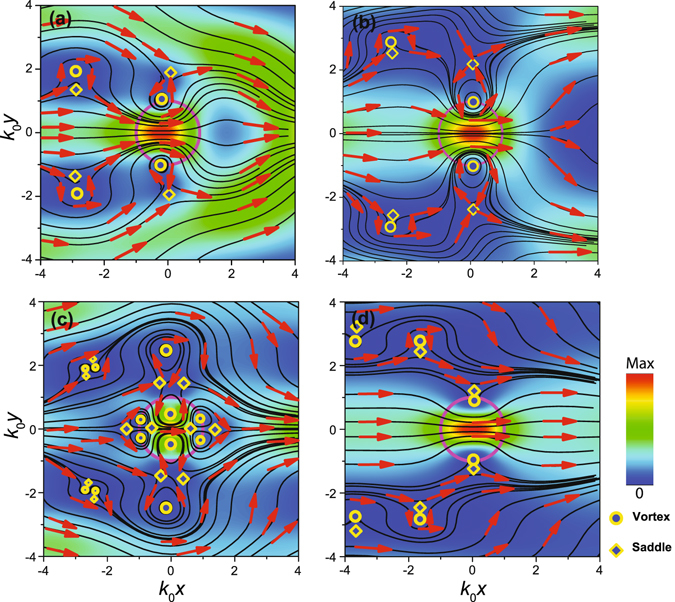
Poynting vector maps for (**a**) *ε*′ = 6, *α* = 60° (optical force ${f}_{x}{k}_{0}/|{A}^{2}|=-0.156$), (**b**) *ε*′ = 6, *α* = 35° $({f}_{x}{k}_{0}/|{A}^{2}|=0.183)$, (**c**) *ε*′ = 14.5, *α* = 60° $({f}_{x}{k}_{0}/|{A}^{2}|=0.101)$ and (**d**) *ε*′ = 14.5, *α* = 35° $({f}_{x}{k}_{0}/|{A}^{2}|=-0.015)$. Colour and arrows indicate the magnitude |**S**| and direction **S**/|**S**| of the Poynting vector, respectively. Parameters: *μ* = 1, *ε*″ = 0, *k*_0_ *R* = 1, *B* = 0.

Figure 4 illustrates the behaviour of Poynting vector **S** in the near-field zone of the cylinder. The maps (a), (b) and (d) are similar, because they show interaction of the incident field with the two multipole moments, electric and magnetic dipoles in (a) and (b) and magnetic dipole and quadrupole in (d). If the quadrupole and both dipole moments are comparable, the Poynting vector distribution is more complicated. Instead of the vortex-saddle couples of singular points one observes the sets of four points, Fig. 4(c). Obviously, the grouping of the singularities to quartets is the indication of the equal excitation of the moments. Keeping in mind that **S** is composed of both incident and scattered fields, the Poynting vector can be presented as the sum of the Poynting vectors of the incident and scattered beams and the interference term: **S** = **S**_*inc*_ + **S**_*sc*_ + **S**_*int*_. In Fig. 4(a) the energy flux density maximum inside the cylinder is shifted to the light source (to the left-hand side). To ensure the force is negative, the backscattering should be smaller than the forward scattering, i.e. the interference term for backward scattering is greater than that for the forward scattering. Then the enhanced forward scattering would push back the particle in accordance with the momentum conservation. Behaviour of the Poynting vector in the case of the Bessel beams scattered by dipole spherical particles possesses the similar features^34^.

Now we shall analytically obtain the range of incident angles required to pull a cylindrical particle. We stress that this range is not sufficient to have the negative force. The analytical expression for the time-averaged optical force exerting on the dipole-quadrupole cylinder follows from the substitution of the scattered electromagnetic fields up to quadrupole terms into the Maxwell stress tensor and subsequent integration over the surface at infinity (see Methods). In the case of the nondiffractive light field of the form ${\bf{E}}(x,y)={\bf{e}}(y)\,\exp ({\rm{i}}\beta {k}_{0}x)$ we get

6 $$\begin{array}{rcl}{f}_{x} & = & \frac{{k}_{0}\beta }{2}{\rm{Im}}({\bf{p}}{{\bf{E}}}^{\ast }+{\bf{m}}{{\bf{H}}}^{\ast }+\frac{1}{2}{{\bf{e}}}_{y}{\hat{q}}_{e}\frac{\partial {{\bf{E}}}^{\ast }}{\partial y}+\frac{1}{2}{{\bf{e}}}_{y}{\hat{q}}_{m}\frac{\partial {{\bf{H}}}^{\ast }}{\partial y})\\  &  & -\frac{{\beta }^{2}{k}_{0}^{2}}{4}{\rm{Re}}({{\bf{e}}}_{x}{\hat{q}}_{e}{{\bf{E}}}^{\ast }+{{\bf{e}}}_{x}{\hat{q}}_{e}{{\bf{E}}}^{\ast })-\frac{\pi {k}_{0}^{3}}{2}{\rm{Re}}({\bf{p}}\times {{\bf{m}}}^{\ast }){{\bf{e}}}_{x}\\  &  & -\frac{\pi {k}_{0}^{4}}{8}{\rm{Im}}({{\bf{e}}}_{x}{\hat{q}}_{e}{{\bf{p}}}^{\ast }+{{\bf{e}}}_{x}{\hat{q}}_{m}{{\bf{m}}}^{\ast }).\end{array}$$

Since the optical forces in the particular cases of TE and TM polarizations of the incident beam have the similar structure, we consider only one polarization to derive the required ranges of angles *α*. Introducing the dipole and quadrupole moments into (6) one writes for the TE-polarized beam (see Methods)

7 $$\begin{array}{rcl}{f}_{x} & = & \frac{{k}_{0}\beta }{2}[{\rm{Im}}{\alpha }_{e}^{\parallel }+{\beta }^{2}{\rm{Im}}{\alpha }_{m}^{\perp }-\frac{{k}_{0}}{2}{(2{\beta }^{2}-1)}^{2}{\rm{Re}}{m}_{xy}-\pi {k}_{0}^{2}{\rm{Re}}({\alpha }_{e}^{\parallel }{\alpha }_{m}^{\perp \ast })\\  &  & -\,\frac{\pi {k}_{0}^{3}}{4}(2{\beta }^{2}-1)\,{\rm{Im}}({\alpha }_{m}^{\perp \ast }{m}_{xy})]{|{E}_{z}|}^{2}.\end{array}$$

The pulling force (7) for dipolar particles possessing *m*_*xy*_ = 0 is realized, if inequality

8 $${\rm{Im}}{\alpha }_{e}^{\parallel }+{\beta }^{2}{\rm{Im}}{\alpha }_{m}^{\perp }-\pi {k}_{0}^{2}{\rm{Re}}({\alpha }_{e}^{\parallel }{\alpha }_{m}^{\perp \ast }) < 0$$

holds. We shall rewrite it in the conditional form for *β* = cos *α* as

9 $${\beta }^{2} < \frac{\pi {k}_{0}^{2}{\rm{Re}}({\alpha }_{e}^{\parallel }{\alpha }_{m}^{\perp \ast })}{{\rm{Im}}{\alpha }_{m}^{\perp }}-\frac{{\rm{Im}}{\alpha }_{e}^{\parallel }}{{\rm{Im}}{\alpha }_{m}^{\perp }}.$$

The recoil force proportional to the dipoles interaction term ${\rm{Re}}({\alpha }_{e}^{\parallel }{\alpha }_{m}^{\perp \ast })$ determines the appearance of the pulling force. The recoil force takes the greatest value, when both polarizabilities are simultaneously maximized, i.e. the Mie coefficients are *b*_0_ = *b*_1_ = 1. Thus, one gets

10 $${\beta }^{2} < \frac{\pi {k}_{0}^{2}(\mathrm{2/(}\pi {k}_{0}^{2}{)}^{2})}{\mathrm{2/}\pi {k}_{0}^{2}}-\frac{\mathrm{1/}\pi {k}_{0}^{2}}{\mathrm{2/}\pi {k}_{0}^{2}} < \frac{1}{2}.$$

The longitudinal wavenumber *β* should be less than $1/\sqrt{2}$; therefore, angle *α* should be greater than 45°. It should be noted that the limiting angle obtained for dipole cylinders is smaller than that for spherical beads (*α* = 60°)^7, 30^.

The first three terms in equation (7) are positive for dipole-quadrupole cylinders, because ${\rm{Im}}({\alpha }_{e}^{\parallel }) > 0$, ${\rm{Im}}({\alpha }_{m}^{\perp }) > 0$ and $-{\rm{Re}}({m}_{xy}) > 0$. The fourth and fifth terms in equation (7) represent the recoil force that can be negative at some conditions. The first recoil term (interaction of electric and magnetic dipoles) is negative only if ${\rm{Re}}({\alpha }_{e}^{\parallel }{\alpha }_{m}^{\perp \ast }) > 0$. The second recoil term (interaction of magnetic dipole and quadrupole moments) may result in pulling the cylinder, if (i) ${\rm{Im}}({\alpha }_{m}^{\perp \ast }{m}_{xy}) < 0$ for $\beta  < 1/\sqrt{2}$ and (ii) ${\rm{Im}}({\alpha }_{m}^{\perp \ast }{m}_{xy}) > 0$ for $\beta  > 1/\sqrt{2}$. Since case (i) yields the same range of *β* as that for the dipole cylinders, we consider only case (ii) presented below as an inequality

11 $$a{\beta }^{4}+b{\beta }^{2}+c < 0,$$

where

12 $$\begin{array}{rcl}a & = & -2{k}_{0}{\rm{Re}}{m}_{xy} > 0,\\ b & = & 2{k}_{0}{\rm{Re}}{m}_{xy}+{\rm{Im}}{\alpha }_{m}^{\perp }-\frac{\pi {k}_{0}^{3}}{2}{\rm{Im}}({\alpha }_{m}^{\perp \ast }{m}_{xy}),\\ c & = & -\frac{{k}_{0}{\rm{Re}}{m}_{xy}}{2}+{\rm{Im}}{\alpha }_{e}^{\parallel }+\frac{\pi {k}_{0}^{3}}{4}{\rm{Im}}({\alpha }_{m}^{\perp \ast }{m}_{xy})-\pi {k}_{0}^{2}{\rm{Re}}({\alpha }_{e}^{\parallel }{\alpha }_{m}^{\perp \ast }\mathrm{).}\end{array}$$

Inequality (11) holds true, if *β* is between the positive roots of the biquadratic equation $a{\beta }_{\pm }^{4}+b{\beta }_{\pm }^{2}+c=0$:

13 $${\beta }_{\pm }=\sqrt{-\frac{b}{2a}\pm \sqrt{{(\frac{b}{2a})}^{2}-\frac{c}{a}}}$$

or

14 $${\beta }_{\pm }=\sqrt{\frac{1}{2}+u(1\pm \sqrt{1+v})},$$

where

15 $$v=\frac{2a}{{(b+a)}^{2}}[2{\rm{Im}}{\alpha }_{m}^{\perp }-\pi {k}_{0}^{3}{\rm{Im}}({\alpha }_{m}^{\perp \ast }{m}_{xy})-2({\rm{Im}}{\alpha }_{e}^{\parallel }+{\rm{Im}}{\alpha }_{m}^{\perp }/2-\pi {k}_{0}^{2}{\rm{Re}}({\alpha }_{e}^{\parallel }{\alpha }_{m}^{\perp \ast }))]\le 0,$$

because for *β* ^2^ > 1/2

16 $${\rm{Im}}{\alpha }_{e}^{\parallel }+{\rm{Im}}{\alpha }_{m}^{\perp }/2-\pi {k}_{0}^{2}{\rm{Re}}({\alpha }_{e}^{\parallel }{\alpha }_{m}^{\perp \ast }) > 0$$

and at the magnetic quadrupolar resonance *b*_2_ = 1 (quantity ${m}_{xy}=-4/\pi {k}_{0}^{3}$)

17 $$2{\rm{Im}}{\alpha }_{m}^{\perp }-\pi {k}_{0}^{3}{\rm{Im}}({\alpha }_{m}^{\perp \ast }{m}_{xy})=-2{\rm{Im}}{\alpha }_{m}^{\perp } < 0.$$

The resonant condition is necessary to provide the strongest recoil force as it was done for the dipolar cylinders. To catch all possible values of *β* suitable for dragging cylinders by light we need to consider the widest range of *β*_−_ < *β* < *β*_+_. The widest range of *β* is achieved for the maximum achievable *v*, i.e. for *v* = 0.

Quantity

18 $$u=\frac{\pi {k}_{0}^{3}{\rm{Im}}({\alpha }_{m}^{\perp \ast }{m}_{xy})-2{\rm{Im}}{\alpha }_{m}^{\perp }}{-8{k}_{0}{\rm{Re}}{m}_{xy}}$$

in equation (14) is maximized in the case of the greatest recoil force, when Mie coefficients *b*_1_ and *b*_2_ turn to the unity resulting in ${m}_{xy}=-4/\pi {k}_{0}^{3}$ and ${\alpha }_{m}^{\perp }=2/\pi {k}_{0}^{2}$. Then

19 $$u=\frac{{\rm{Im}}{\alpha }_{m}^{\perp }}{-4{k}_{0}{\rm{Re}}{m}_{xy}}=\frac{1}{8}$$

and *β* is confined by

20 $$\frac{1}{\sqrt{2}} < \beta  < \frac{\sqrt{3}}{2},$$

i.e. incident angles are within 30° < *α* < 45°. Analytically derived *α* for dipole and dipole-quadrupole cylindrical particles fully support previous conclusions grounded on the numerical calculations.

**Figure 5:**
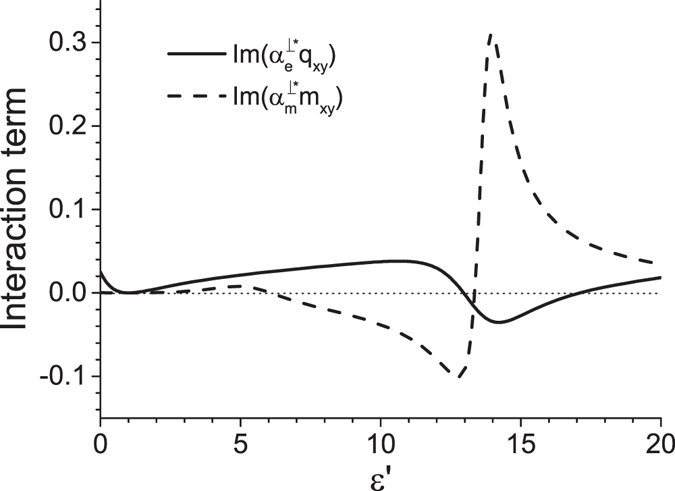
Electric and magnetic dipole-quadrupole-interaction terms, ${\rm{Im}}({\alpha }_{e}^{\perp \ast }{q}_{xy})$ and ${\rm{Im}}({\alpha }_{m}^{\perp \ast }{m}_{xy})$, as function of dielectric permittivity *ε*′. Parameters: *μ* = 1, *ε*″ = 0, *k*_0_ *R* = 1.

When nonmagnetic cylindrical particles are illuminated by a TM-polarized beam (*A* = 0 in equation (1)), the negative force due to the interaction of dipoles exists, but the pulling force due to the interaction of electric dipole and electric quadrupole does not. There is no contradiction with the derived range of angles 30° < *α* < 45°, because this inequality is not the sufficient condition for the emergence of an optical pulling force. To show the reason of vanishing of the quadrupolar pulling force we plot the terms of dipole-quadrupole interaction in Fig. 5. The TE-polarized light beam yields much stronger recoil force than the TM-wave, therefore, the pulling is available for the TE polarization only. So, controlling the polarization of the incident wave can be used for the switching direction of the optical force in the quadrupolar regime. Noting that the Mie coefficients flip, when the dielectric permittivity and magnetic permeability interchange, one concludes that the TM-polarized beam illuminating magnetic cylindrical particles (*ε* = 1) introduces the same force as the TE-polarized beam incident on dielectric cylinders (*μ* = 1).

## Discussion

In general, nonparaxial light beams with continuous propagation invariance have to be used for pulling both spherical and cylindrical particles, although the nonparaxial condition for dragging cylindrical particles is less stringent being *α* > 45° and 30° < *α* < 45° in dipolar and quadrupolar approximations, respectively. Curiously, particle’s geometrical shape plays an important part in changing the minimum angle *α*, which is 60° for three-dimensional spherical particles^7, 30^ and 45° for two-dimensional cylinders in the dipolar approximation. Since a prolate spheroidal form is something intermediate between the spherical and cylindrical ones, the minimum angle *α* in this case is expected to be between 45° and 60°. Concave particles having rather hyperboloid-like than spheroid-like form may give us the opportunity for further reduction of the minimum *α*.

The optical pulling forces exerting on cylindrical particles in the quadrupolar approximation are caused by the dipole-quadrupole recoil force. Theoretically it is possible to reduce angle *α* down to 30°, but with costs associated with the thorough control on the system parameters. In principle, one may move further towards the paraxial regime of the incident light beams, if the recoil force is guaranteed by the higher-order multipoles. However their resonant response is narrow and can be easily suppressed by the presence of material losses. That is why the decrease of *α* below 30° is questionable. The dipole-quadrupole optical pulling force is known for dielectric spherical particles as well^1, 2^.

In this article we consider the idealized geometry, when the cylinder position is fixed with respect to the incident beam. But position of a free-standing cylinder is not stable: it should be exerted by the optical torque^35^. Nevertheless, we expect that the results obtained in this article could be exploited in a periodic metamaterial with dielectric cylinders as meta-atoms. Then there are no problems with the illumination of the metamaterial in a proper manner. We can anticipate that the whole metamaterial can be pulled by the light beam, if a single meta-atom is pulled. According to work^36^ the optical force acting on a finite metamaterial is not defined by its microstructure and, therefore, the effective medium approximation can be employed. Homogenizing a nanorod metamaterial as a dipole-quadrupole continuum^37, 38^ one may potentially pull it using incident light.

## Methods

### Derivation of the dipole and quadrupole moments

We find multipole moments by comparing the magnetic fields in the far-field zone excited by the multipoles and scattered by a couple of plane waves (1). Considering the infinite cylindrical particle as a line of homogeneously distributed point dipoles and quadrupoles^1, 39^, we denote the linear densities of the electric dipoles, magnetic dipoles, electric quadrupoles and magnetic quadrupoles as **p**, **m**, ${\hat{q}}_{e}$ and ${\hat{q}}_{m}$, respectively.

Then from the vector potential in the far-field zone

21 $${{\bf{A}}}^{(d-q)}(\rho ,\phi )=i\sqrt{2\pi }\frac{{e}^{i\omega \rho /c-i\pi \mathrm{/4}}}{c\sqrt{\omega \rho /c}}[-i\omega {\bf{p}}+\frac{i\omega }{c}({{\bf{e}}}_{\rho }\times {\bf{m}})-\frac{{\omega }^{2}}{2c}{\hat{q}}_{e}{{\bf{e}}}_{\rho }+\frac{{\omega }^{2}}{2{c}^{2}}{{\bf{e}}}_{\rho }\times {\hat{q}}_{m}{{\bf{e}}}_{\rho }],$$

we write the magnetic field in free space ${\bf{H}}={\bf{B}}=\nabla \times {\bf{A}}={{\bf{e}}}_{\rho }\times \partial {\bf{A}}/\partial \rho$ or, explicitly,

22 $${{\bf{H}}}^{(d-q)}(\rho ,\phi )=i\sqrt{2\pi }{k}_{0}^{2}\frac{{e}^{i{k}_{0}\rho -i\pi \mathrm{/4}}}{\sqrt{{k}_{0}\rho }}[{{\bf{e}}}_{\rho }\times {\bf{p}}^{\prime} +{{\bf{e}}}_{\rho }\times ({\bf{m}}^{\prime} \times {{\bf{e}}}_{\rho })],$$

where *ρ*, *φ* and *z* are the cylindrical coordinates, **e**_*ρ*_, **e**_*φ*_ and **e**_*z*_ are the basis vectors. Effective dipole moments are equal to

23 $${\bf{p}}^{\prime} ={\bf{p}}-\frac{i{k}_{0}}{2}{\hat{q}}_{e}{{\bf{e}}}_{\rho },\,{\bf{m}}^{\prime} ={\bf{m}}-\frac{i{k}_{0}}{2}{\hat{q}}_{m}{{\bf{e}}}_{\rho }.$$

On the other hand, the magnetic field scattered by a dipole-quadrupole cylinder, i.e. up to the Mie coefficients *a*_2_ and *b*_2_, is of the form^31^

24 $$\begin{array}{rcl}{{\bf{H}}}_{PW}^{(s)} & = & \sqrt{\frac{2}{\pi {k}_{0}\rho }}{{\rm{e}}}^{{\rm{i}}{k}_{0}\rho -{\rm{i}}\pi \mathrm{/4}}[{a}_{0}{c}_{1}{{\bf{e}}}_{z}+{b}_{0}{c}_{2}{{\bf{e}}}_{\phi }-2\,\cos \,\phi ({a}_{1}{c}_{1}{{\bf{e}}}_{z}+{b}_{1}{c}_{2}{{\bf{e}}}_{\phi })\\  &  & +\,2\,\cos \,\mathrm{(2}\phi )({a}_{2}{c}_{1}{{\bf{e}}}_{z}+{b}_{2}{c}_{2}{{\bf{e}}}_{\phi })]\end{array}$$

in the case of the incident plane wave

25 $${{\bf{E}}}^{(inc)}(x)={{\rm{e}}}^{{\rm{i}}{k}_{0}x}({c}_{1}{{\bf{e}}}_{y}+{c}_{2}{{\bf{e}}}_{z}).$$

when the direction of an incident plane wave in the cross-section of the cylinder is oriented at angle *α* with respect to the axis *x*, the unit vectors **e**_*z*_ and **e**_*φ*_ do not change, but angle *φ* should be replaced with *φ* − *α* in equation (24). Let us have a couple of plane waves now, the fields of which are given by (1). The angle of incidence for the first wave equals *α*, while its electric field (25) is characterized by the amplitudes ${c}_{1}^{^{\prime} }$ and ${c}_{2}^{^{\prime} }$. For the second wave the incident angle and amplitudes are −*α*, ${c}_{1}^{^{\prime\prime} }$ and ${c}_{2}^{^{\prime\prime} }$. The scattered magnetic field ${{\bf{H}}}^{(s)}={{\bf{H}}}_{PW}^{(s)}(\rho ,\phi -\alpha )+{{\bf{H}}}_{PW}^{(s)}(\rho ,\phi +\alpha )$ for the particular case ${c}_{1}^{^{\prime} }={c}_{1}^{^{\prime\prime} }=B/2$ and ${c}_{2}^{^{\prime} }={c}_{2}^{^{\prime\prime} }=A/2$ (*A* and *B* are defined by equation (1)) is equal to

26 $$\begin{array}{rcl}{{\bf{H}}}^{(s)} & = & \sqrt{\frac{2}{\pi {k}_{0}\rho }}{{\rm{e}}}^{{\rm{i}}{k}_{0}\rho -{\rm{i}}\pi \mathrm{/4}}[{a}_{0}B{{\bf{e}}}_{z}+{b}_{0}A{{\bf{e}}}_{\phi }-2\,\cos \,\alpha \,\cos \,\phi ({a}_{1}B{{\bf{e}}}_{z}+{b}_{1}A{{\bf{e}}}_{\phi })\\  &  & +\,2\,\cos \,\mathrm{(2}\alpha )\,\cos \,\mathrm{(2}\phi )({a}_{2}B{{\bf{e}}}_{z}+{b}_{2}A{{\bf{e}}}_{\phi })].\end{array}$$

Comparing magnetic fields (22) and (26) it is straightforward to derive the electric and magnetic dipole and quadrupole moments (2).

In general, the quadrupole moments of a cylindrical particle are the nondiagonal traceless matrices that can be written for any incident electromagnetic field as

27 $${\hat{q}}_{e}=\frac{{q}_{xy}}{i{k}_{0}}{I}_{z}(\nabla \otimes {\bf{E}}+{(\nabla \otimes {\bf{E}})}^{T}){I}_{z},\,{\hat{q}}_{m}=\frac{{m}_{xy}}{i{k}_{0}}{I}_{z}(\nabla \otimes {\bf{H}}+{(\nabla \otimes {\bf{H}})}^{T}){I}_{z},$$

where the superscript *T* denotes the matrix transpose and ${I}_{z}={{\bf{e}}}_{x}\otimes {{\bf{e}}}_{x}+{{\bf{e}}}_{y}\otimes {{\bf{e}}}_{y}$ is the projection operator onto the plane of cylinder’s cross-section (*x*, *y*).

### Optical forces exerting dipole-quadrupole cylinders

An optical force can be divided into two parts, the first of which, **F**_*inc*_, originates from the interaction of the incident electromagnetic field with the induced multipoles, while the second force, **F**_*mult*_, stems from the interaction of the fields of multipoles with the multipoles themselves^40^. The latter force is also called a recoil force.

Force **F**_*inc*_ is defined by the whole multipole moments **p** *l*, **m** *l*, ${\hat{q}}_{e}l$ and ${\hat{q}}_{m}l$ (*l* is the length of the cylinder) as follows

28 $${{\bf{F}}}_{inc}=\frac{1}{2}{\rm{Re}}(\nabla ({\bf{p}}l\mathop{{{\bf{E}}}^{\ast }}\limits^{\downarrow })+\nabla ({\bf{m}}l\mathop{{{\bf{H}}}^{\ast }}\limits^{\downarrow }))+\frac{1}{4}{\rm{Re}}(\nabla (\nabla {\hat{q}}_{e}l\mathop{{{\bf{E}}}^{\ast }}\limits^{\downarrow })+\nabla (\nabla {\hat{q}}_{m}l\mathop{{{\bf{H}}}^{\ast }}\limits^{\downarrow })),$$

where differential operator $\nabla$ acts only on the incident fields (the quantities subject to differentiation are denoted with an arrow on top), e.g., as ${[\nabla ({\bf{p}}\mathop{{{\bf{E}}}^{\ast }}\limits^{\downarrow })]}_{i}=\frac{\partial }{\partial {x}_{i}}({\sum }_{j=1}^{3}{p}_{j}\mathop{{E}_{j}^{\ast }}\limits^{\downarrow })={\sum }_{j=1}^{3}{p}_{j}\frac{\partial {E}_{j}^{\ast }}{\partial {x}_{i}}$. Incident fields ${\bf{E}}\equiv {{\bf{E}}}^{(inc)}$ and ${\bf{H}}\equiv {{\bf{H}}}^{(inc)}$ and their derivatives are calculated in the centre of the particle *ρ* = 0.

Since the distributions of the multipole fields depend on particle’s shape, recoil force **F**_*mult*_ is geometry dependent. A proper relationship follows from the integration of the scattered dipole-quadrupole fields at infinity as (see the paper^41^ for spherical particles)

29 $$\begin{array}{rcl}{{\bf{F}}}_{mult} & = & -\frac{1}{16\pi }{\int }_{{S}_{\infty }}({|{{\bf{E}}}^{(s)}|}^{2}+{|{{\bf{H}}}^{(s)}|}^{2}){{\bf{e}}}_{\rho }ds\\  & = & -\frac{1}{8\pi }{\int }_{S}{|{{\bf{H}}}^{(s)}|}^{2}{{\bf{e}}}_{\rho }ds\\  & = & -\frac{{k}_{0}^{3}l}{4}{\int }_{0}^{2\pi }{|{{\bf{e}}}_{\rho }\times {\bf{p}}^{\prime} +({{\bf{e}}}_{\rho }\times {\bf{m}}^{\prime} )\times {{\bf{e}}}_{\rho }|}^{2}{{\bf{e}}}_{\rho }d\phi \\  & = & -\frac{\pi {k}_{0}^{3}l}{2}{\rm{Re}}({\bf{p}}\times {{\bf{m}}}^{\ast })-\frac{\pi {k}_{0}^{4}l}{8}{\rm{Im}}({\hat{q}}_{e}{{\bf{p}}}^{\ast }+{\hat{q}}_{m}{{\bf{m}}}^{\ast }).\end{array}$$

Using the derived expressions for **F**_*inc*_ and **F**_*mult*_ the optical force per unit cylinder’s length takes the form

30 $$\begin{array}{rcl}{\bf{f}} & = & \frac{{{\bf{F}}}_{inc}+{{\bf{F}}}_{mult}}{l}\\  & = & \frac{1}{2}{\rm{Re}}(\nabla ({\bf{p}}\mathop{{{\bf{E}}}^{\ast }}\limits^{\downarrow })+\nabla ({\bf{m}}\mathop{{{\bf{H}}}^{\ast }}\limits^{\downarrow }))+\frac{1}{4}{\rm{Re}}(\nabla (\nabla {\hat{q}}_{e}\mathop{{{\bf{E}}}^{\ast }}\limits^{\downarrow })+\nabla (\nabla {\hat{q}}_{m}\mathop{{{\bf{H}}}^{\ast }}\limits^{\downarrow }))\\  &  & -\frac{\pi {k}_{0}^{3}}{2}{\bf{p}}\times {{\bf{m}}}^{\ast }-\frac{\pi {k}_{0}^{4}}{8}{\rm{Im}}({\hat{q}}_{e}{{\bf{p}}}^{\ast }+{\hat{q}}_{m}{{\bf{m}}}^{\ast }).\end{array}$$

Let us consider a nondiffractive TE-polarized beam (1). Since *B* = 0 in equation (1), the electric and magnetic fields equal **E** = *E*_*z*_ **e**_*z*_ and **H** = *H*_*x*_ **e**_*x*_ + *H*_*y*_ **e**_*y*_, respectively. When calculated at the origin of the coordinate system, the magnetic field reduces to **H** = *H*_*y*_ **e**_*y*_ = −*βE*_*z*_ **e**_*y*_. Partial derivative required in equation (6) reads $\partial {\bf{H}}/\partial y=i{k}_{0}(\beta {H}_{y}+{E}_{z}){{\bf{e}}}_{x}$. The dipole and quadrupole moments for the TE-polarized beam are equal to ${\bf{p}}={\alpha }_{e}^{\parallel }{\bf{E}}$, ${\bf{m}}={\alpha }_{m}^{\perp }{\bf{H}}$, ${\hat{q}}_{e}=0$, and ${\hat{q}}_{m}=[\mathrm{(2}{\beta }^{2}-\mathrm{1)}/\beta ]{m}_{xy}{H}_{y}({{\bf{e}}}_{x}\otimes {{\bf{e}}}_{y}+{{\bf{e}}}_{y}\otimes {{\bf{e}}}_{x})$. Note that the responses for the given polarization are determined by Mie coefficients *b*. With the above quantities in mind, the force along the optical axis is given by equation (7).

TM-polarization is characterized by *A* = 0 in equation (1), that is, electric and magnetic fields are equal to **E** = *E*_*x*_ **e**_*x*_ + *E*_*y*_ **e**_*y*_ and **H** = *H*_*z*_ **e**_*z*_, respectively. The electric field in the origin *ρ* = 0 is **E** = *E*_*y*_ **e**_*y*_ = *βH*_*z*_ **e**_*y*_, while the derivative takes the form $\partial {\bf{E}}/\partial y=i{k}_{0}(\beta {E}_{y}-{H}_{z}){{\bf{e}}}_{x}$. The dipole and quadrupole moments are determined by Mie coefficients *a* as ${\bf{p}}={\alpha }_{e}^{\perp }{\bf{E}}$, ${\bf{m}}={\alpha }_{m}^{\parallel }{\bf{H}}$, ${\hat{q}}_{e}=\mathrm{[(2}{\beta }^{2}-\mathrm{1)}/\beta ]{q}_{xy}{E}_{y}({{\bf{e}}}_{x}\otimes {{\bf{e}}}_{y}+{{\bf{e}}}_{y}\otimes {{\bf{e}}}_{x})$, and ${\hat{q}}_{m}=0$. Then the optical force component *f*_*x*_ reads

31 $$\begin{array}{rcl}{f}_{x} & = & \frac{{k}_{0}\beta }{2}[{\beta }^{2}{\rm{Im}}{\alpha }_{e}^{\perp }+{\rm{Im}}{\alpha }_{m}^{\parallel }-\frac{{k}_{0}}{2}{\mathrm{(2}{\beta }^{2}-\mathrm{1)}}^{2}{\rm{Re}}{q}_{xy}-\pi {k}_{0}^{2}{\rm{Re}}({\alpha }_{e}^{\perp }{\alpha }_{m}^{\parallel \ast })\\  &  & -\frac{\pi {k}_{0}^{3}}{4}\mathrm{(2}{\beta }^{2}-1)\,{\rm{Im}}({\alpha }_{e}^{\perp \ast }{q}_{xy})]{|{H}_{z}|}^{2}.\end{array}$$
